# Acrylamide in Corn-Based Thermally Processed Foods: A Review

**DOI:** 10.1021/acs.jafc.1c07249

**Published:** 2022-03-31

**Authors:** Slađana Žilić, Valentina Nikolić, Burçe Ataç Mogol, Aytül Hamzalıoğlu, Neslihan Göncüoğlu Taş, Tolgahan Kocadağlı, Marijana Simić, Vural Gökmen

**Affiliations:** † Maize Research Institute, Group of Food Technology and Biochemistry, Slobodana Bajića 1, 11185 Belgrad- Zemun, Serbia; ‡ Food Quality and Safety (FoQuS) Research Group, Department of Food Engineering, Hacettepe University, 06800 Beytepe, Ankara, Turkey

**Keywords:** acylamide, corn-based foods, thermal processing, asparagine, reducing sugars, benchmark levels

## Abstract

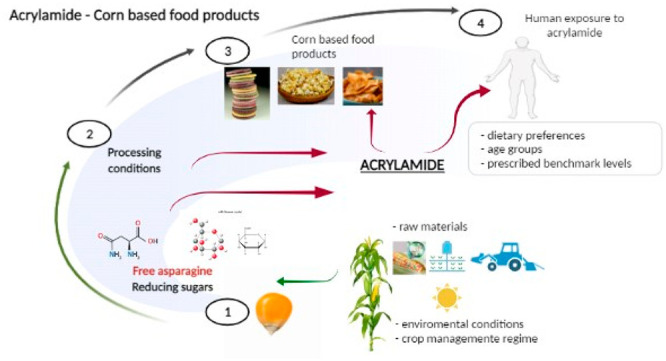

Widely consumed thermally processed corn-based foods can have a great contribution to acrylamide dietary intake, thus bearing a high public health risk and requiring attention and application of strategies for its reduction. This paper reviews the literature on the acrylamide content of corn-based food products present in the market around the world. The potential of corn for acrylamide formation due to its content of free asparagine and reducing sugars is described. Human exposure to acrylamide from corn-based foods is also discussed. The content of acrylamide in corn/tortilla chips, popcorn, and corn flakes, as widely consumed products all over the world, is reported in the literature to be between 5 and 6360 μg/kg, between <LOD and 2220 μg/kg and between <LOD and 1186 μg/kg, respectively. Although these products are important acrylamide sources in the common diet of all age populations, higher intake values occurred among younger generations.

## Introduction

Through the centuries, corn has been representing a product, food, fodder, merchandise, firewood, fuel, construction material, industrial raw material, and medicinal and decorative plant for many civilizations and nations. Corn plays an important role in food production today. A wide range of corn-based food products such as different bakery products, snack foods, cakes and cookies, breakfast cereals, porridges, beverages, etc. can be processed from corn grain, flour, or starch at home on a small local scale, as well as on a larger industrial scale. However, the transformation of the raw corn grain into food products is mostly accompanied by a thermal treatment that can result in the formation of processing contaminants such as acrylamide. Acrylamide (prop-2-enamide) is a well-known industrial chemical that, based on its carcinogenic action in rodents, the International Agency for Research on Cancer has classified as probably carcinogenic to humans (Group 2A).^1^ However, Eisenbrand^2^ states that the genotoxicity of acrylamide may rather be understood as an effect occurring, if at all, at exceedingly high dose levels, not relevant to realistic physiological conditions, especially not to those prevailing at consumers’ dietary exposure level. The results of LoPachin and Lehning^3^ showed that exposure to acrylamide caused damage to the nervous system in humans and animals. Acrylamide is also considered a reproductive toxin, with mutagenic and carcinogenic properties in experimental mammalian *in vitro* and *in vivo* systems.^4^ Acrylamide in foods is formed via the Maillard reaction from free asparagine in the presence of carbonyl compounds such as reducing sugars during thermal processes. Therefore, food raw materials rich in both of these precursors, such as cereals, have a high potential for the formation of acrylamide. Basic sources of acrylamide exposure from foodstuffs depend on national/regional food habits. Generally, corn-based food products, such as tortilla chips and breakfast cereals, are widely consumed all over the world. Regarding of consumption of salty snacks, corn chips ranked in the second place behind potato products.^5^ In the area of sweet and savory snacks in the United States, the market of tortilla chips ranked first in terms of sales volume.^6^ Hence, exposure to acrylamide due to the high consumption of corn-based products creates health concerns and raises the need for acrylamide reduction in these products.

In this study, an overview of the results related to the potential of corn grain for acrylamide formation, to the content of acrylamide in corn-based thermally processed foods, as well as to human exposure to acrylamide from corn-based products is presented. The study also presents the benchmark levels for acrylamide defined by the European Commission Regulation 2017/2158/EC^7^ and adopted in many countries worldwide for products that can be prepared, inter alia, entirely from corn flour or based on corn flour. The strategies that are being or could be developed to reduce acrylamide levels in corn derived food products are described as well. A brief overview of data on the production and consumption of corn in the world, as well as on its widely consumed thermally processed food products, is given.

## Corn Production and Corn-Based Food Products

### Worldwide Production and Consumption of Corn

**Figure 1:**
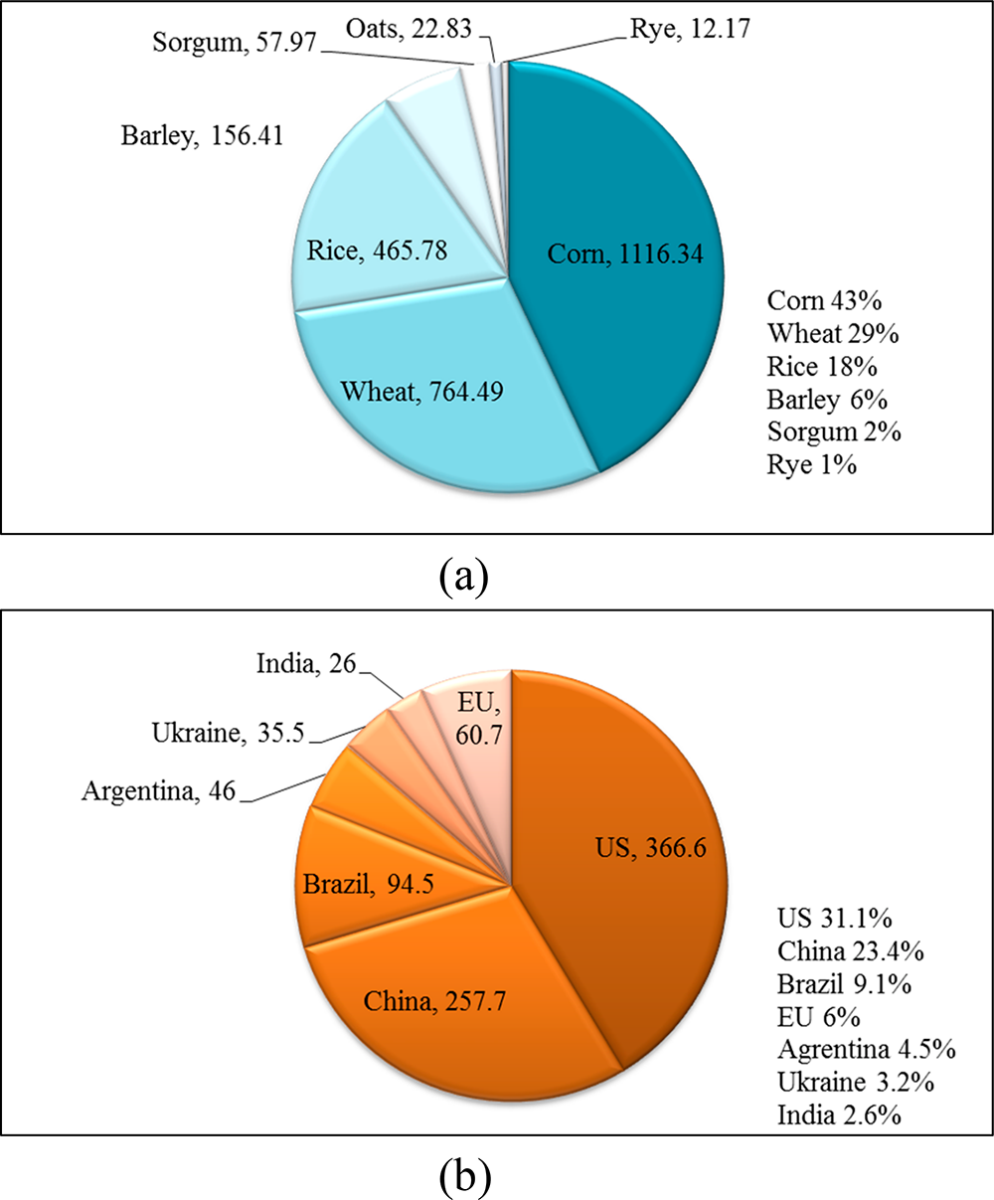
Worldwide production, in 2019/2020, (a) of cereals by type (million tonnes and % of total cereal production)^8^ and (b) of corn by country (million tonnes^7^ and % of total corn production).^10^

**Figure 2:**
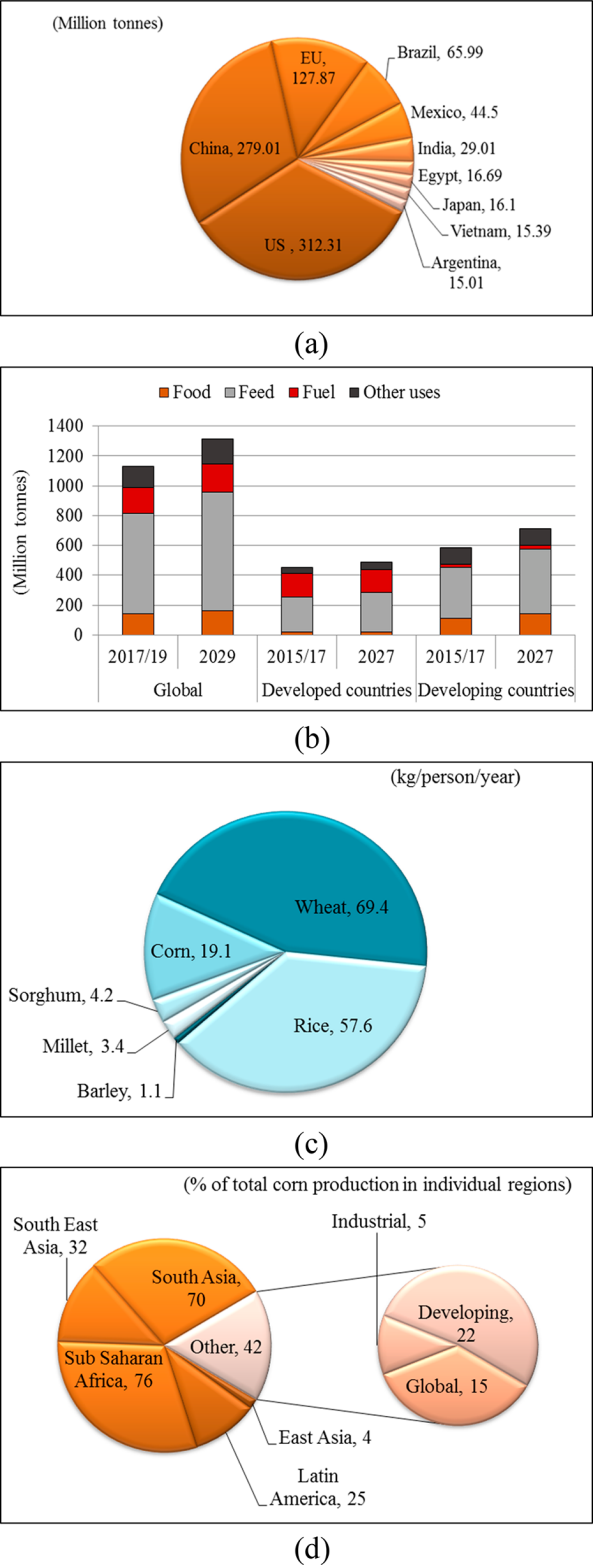
Worldwide consumption of cereals and corn: (a) Consumption of corn by country in 2019/2020 (million tonnes);^11^ (b) consumption of corn by usage area (million tonnes);^12^ (c) consumption of cereals as food in 2000 (kg/person/year);^13^ (d) consumption of corn as food in 2020 by region (% of total corn production in particular regions).^14^

**Figure 3:**
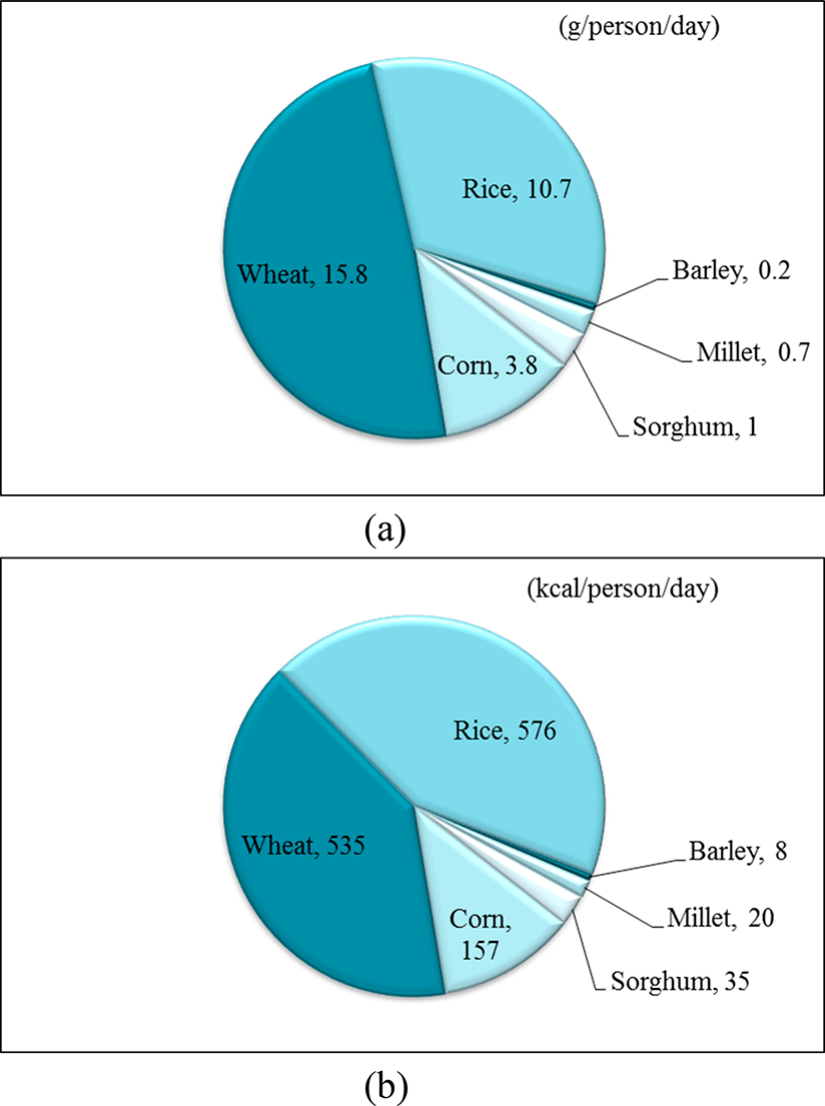
Worldwide consumption of cereals per capita in 2000 (a) as protein (g/person/day) and (b) as energy (kcal/person/day).^13^

Corn is the main cereal, given the volume of production worldwide. With a production volume of around 1.1 billion tonnes and a 43% share in the total world cereal production, corn took the global leadership position in the marketing year 2019/20 (Figure 1a).^8^ In the marketing year 2019/20, the United States alone was responsible for over one-third of global corn production, while together with China it accounted for more than half of the worldwide corn production (Figure 1b). Other significant corn producers are Brazil, Argentina, Ukraine, India, and Mexico. The EU produced 60.7 million tonnes of corn grain, i.e. 6% of the total world corn production, in the marketing year 2019/20 (Figure 1b).^9,10^ The United States is the leading consumer of corn worldwide, followed by China and the EU (Figure 2a).^11^ According to the FAO data, corn grain is a key ingredient in animal feed. Globally, 41% of the total corn production was used on average in the production of animal feed in the three-year period from 2017 to 2019 (Figure 2b).^12^ The corn feed market has been growing especially in countries such as China and India. As a staple food, corn ranks third in the world, after wheat and rice. In 2000, 12% of the total consumption of cereals as food in the world was corn (19.1 kg/person/year) (Figure 2c).^13^ In the three-year period from 2017 to 2019, over 141 million tonnes of corn were used for food production on average. Corn is a dietary staple for millions of people, and it is the most important plant source of food, i.e. nutritional compounds for people in the developing world, especially in Africa, Asia, and Latin America. Industrialized countries used 5% of the total corn production for food in 2020, while developing countries used 22% (Figure 2d).^14^ Globally, corn consumption is expected to increase by 13% from the three-year average (2017–2019) to 2029 (Figure 2b).^12^ Regarding the share of proteins and calories, the role of corn within cereals in human consumption varies significantly across regions. On average, the estimated daily intake of proteins and energy through corn or corn-based food products was 3.8 g/person and 157 kcal/person, respectively, in 2000 (Figure 3).^13^

### Widely Consumed Corn-Based Thermally Processed Foods

**Figure 4:**
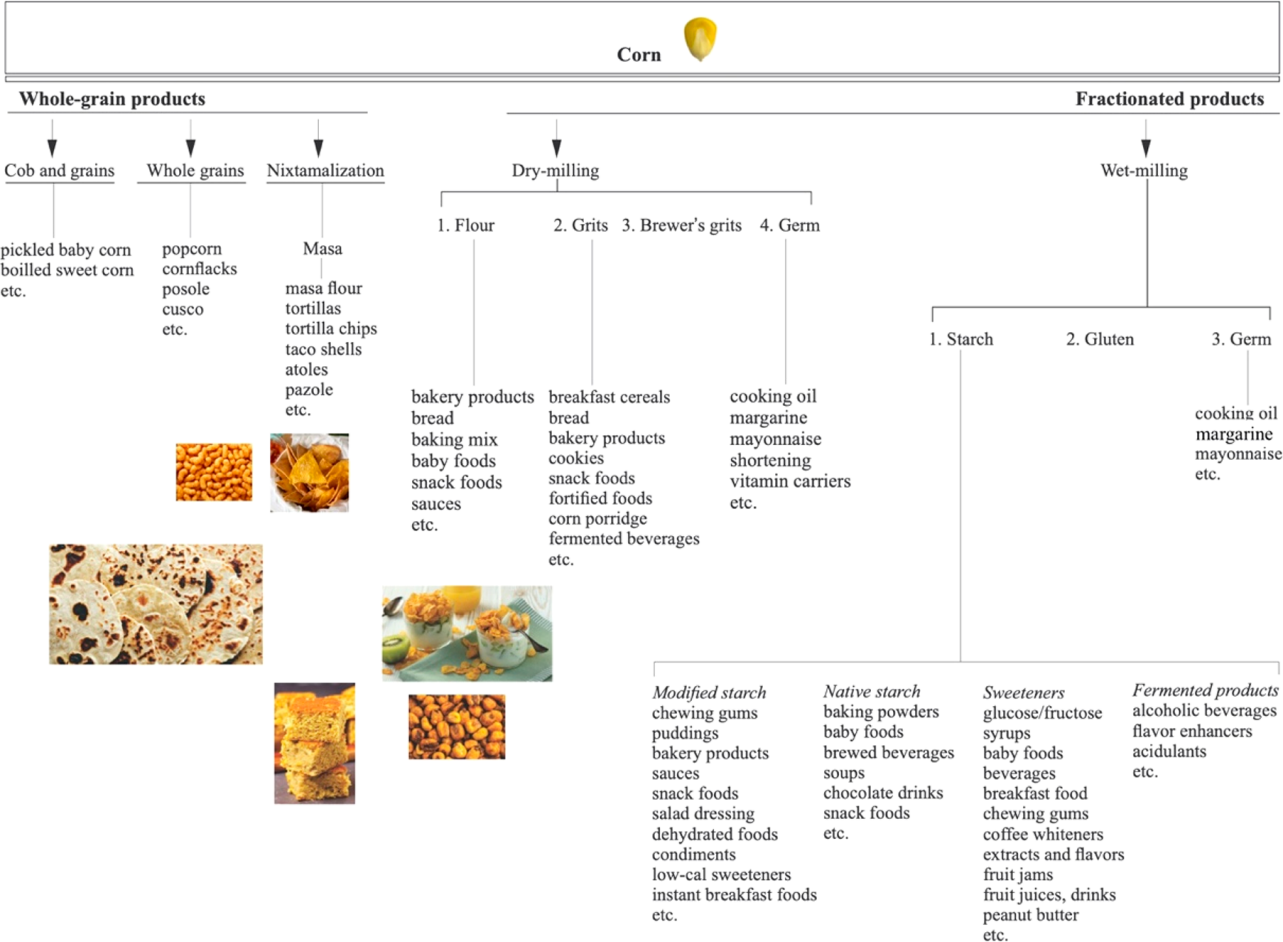
Major unit operations in corn processing and the corn-based food products thereof.

Due to its diverse functionality, corn is a widely used raw material in the food industry. Corn-based food products can be divided into those produced from whole corn grain and those produced from grain fractions by dry- and wet-milling as two basic technological procedures in corn processing (Figure 4). As widely consumed thermally processed corn-based foods, tortillas, tortilla/corn chips, cornflakes, breakfast foods, corn-based bread, cookies, and snacks such as popcorn can have a great contribution to the acrylamide dietary intake, thus bearing a high public health risk. For this reason, a brief overview of the consumption of these products is given as follows.

Tortillas are the main source of energy, protein, and calcium in Mexico, providing 70% of the calories and 90% of the total protein intake.^15^ An average Mexican consumes more than 80 kg of corn tortillas annually. Currently, about 800 million tortillas are consumed per day in Mexico.^16^ Approximately 120 million tortillas are consumed yearly in the United States, making these the second most popular baked product, after white bread. Tortillas currently represent 30% of all baked product sales in the United States.^17^ The global tortilla market accounted for US$ 37.87 billion in 2018 with an expected annual growth rate of 5.2% during the period 2019–2027.^18^ According to the data published by Rooney and Serna-Saldivar,^19^ Mexico accounted for 42% of the world’s production of tortillas in 2012, followed by the United States with 36%, Central America with 9%, and other countries with 13%. The European tortilla market was valued at US$ 4.1 billion in 2018, with the largest markets being the United Kingdom, Spain, The Netherlands, Germany, and France.^20^ Corn tortillas are prepared from the masa/dough after the process of thermal-alkaline cooking of corn with lime (Ca(OH)_2_) and steeping of cooked corn for 12–16 h, and this process is called nixtamalization. Tortillas can be industrially produced by dough pressing (hot-press) and extrusion (die-cut). Cut disks are baked at 177–260 °C for 20 to 50 s. Today, products derived from nixtamalized corn masa such as tortilla chips, corn chips, and taco shells are extensively sold as snack foods. Unlike tortillas, in tortilla chips’ production the thin and shaped nixtamalized masa is baked at 260–290 °C for 35 to 50 s and then fried in oil at a temperature of 170–190 °C, or even 210 °C depending on the type of corn, for 50 to 90 s. The global tortilla chips’ market size was estimated at US$ 21.13 billion in 2019, and it has been expected to reach US$ 22.04 billion in 2020. Due to the presence of several prominent ready-to-eat food brands, North America dominated the tortilla chips’ market with a share of 40.9% in 2019.^21^ Sales of these snacks in the United States in 2004 totaled more than US$ 5 billion^6^ while in 2020 they totaled approximately US$ 6 billion.^22^ The largest tortilla chips’ markets in Europe are those in the United Kingdom and Germany. The largest consumers of tortilla chips in Asia are China, India, and Saudi Arabia, and in South America the largest is Brazil.^21^

According to the data of Statista,^23^ out of the total number of surveyed citizens of the United States, 6.73 million of them eat 8.2 kg or more of cornflakes breakfast cereal in 7 days. Annually, this totals 2.9 billion kg. The surveyed 23.44 million citizens eat 0.82–1.68 kg in 7 days, while 5.97 million eat from 4.1 to 7.38 kg. The most commonly used processes in cornflakes’ production are those of the thermal or hydrothermal type, together with mechanical treatment. Among others, these include extrusion, expansion, and micronization. The usual process consists of mixing materials, extruding, cooling, flaking, drying, toasting, coating with sugar or honey, drying again, and cooling. The cooking or roasting temperature of corn during extrusion and micronization is around 140 °C.^24^

Statistics show that popcorn is the most popular snack in the world. According to data of Statista,^25^ 232.51 million Americans consumed popcorn products in 2020. Americans eat about 14.3 billion liters of popcorn a year. This averages to about 46 L per person. According to popcorn market analysis data reported by 360 Market Updates, the global popcorn market was valued at US$ 3310 million in 2018 and will reach US$ 5550 million by the end of 2025.^26^ According to the same source, the USA revenue of the global popcorn market exceeded 56% in 2016. The USA was followed by Europe. In addition to high temperature, popular toppings or flavors added during microwave and conventional popping can promote the formation of Maillard reaction products in popcorn.^27^

Last but not least, corn flour is also used for making bread and pastries. Although not much attention is paid to this, the use of corn in the diet through its use as a mixture for bread is not negligible from the point of view of acrylamide intake. It was estimated that about 540 million kg of food products were bakery products in the USA in 1996 with formulations that usually contained corn flour from 30% to 50%.^28^ It could be concluded that 60 to 270 million kg of corn flour is consumed annually in the USA for the production of bread. Finally, it should be emphasized that recent findings related to celiac disease, which occurs in approximately 1% of the world population, have led to increased interest in the development of new gluten-free foods, including those based on corn flour. The results of Verardo et al.^29^ are encouraging, as they showed that gluten-free infant cereal formulations, including those with corn, had the lowest content of acrylamide.

## Precursors of Acrylamide in Corn Grain and Flour

It is well-known that the precursors of acrylamide in thermally processed foods are free asparagine (Asn) and reducing sugars. Through decarboxylation and deamination reactions, Asn alone is scarcely converted into acrylamide.^30^ However, when a carbonyl source is present, the yield of acrylamide from Asn is much higher. Yaylayan et al.^30^ reported the acrylamide formation efficiency as the gas chromatographic peak area per mole of starting asparagine generated at 350 °C in different model systems. They found the efficiency as 0 (area/mol) in the asparagine system while it was 4.9 × 10^11^ in the asparagine–glucose, 6.6. × 10^11^ in the asparagine–fructose, and 8.6 × 10^11^ in the asparagine–glyceraldehyde systems, respectively. Given that reducing sugars abound in cereal grains, the concentration of free Asn is the most important or the rate limiting factor in affecting the acrylamide formation in cereal-based foods and, thus, in corn-based food products.

### Free Asparagine

According to Sodek and Wilson,^31^ the total free amino acids have been determined as 4.4% and 2.2% of the total grain nitrogen content in two corn inbred lines and 2.9% in a standard seeded hybrid. The content of total free amino acids in these genotypes was 0.80, 0.44, and 0.47 mg N/g, respectively. Taking into account the content of total free amino acids in other cereals, Lea et al.^32^ concluded that free amino acids generally accounted for about 5% or less of the total nitrogen in cereal grains and Asn accounted for a low proportion (certainly less than 10%) of this fraction. However, these values may be a result to a great extent of both genetic and environmental factors. Harrigan et al.^33^ analyzed the content of free amino acids in the grain of corn hybrids obtained by crossing 48 inbred lines to two testers/parents and grown at three different locations in Iowa. The most abundant free amino acids were proline and then asparagine, aspartic acid, and glutamic acid. Based on statistical analysis, the authors concluded that the content of protein bound amino acids was more susceptible to location effects, whereas the free amino acid levels were, in general, more susceptible to a tester/parent effect, as well as the fact that these two sets of compositional components are under different genetic or regulatory control. Higher mean values of the free Asn content were most pronounced in hybrids derived from the Iodent tester group. The free Asn content in this group of corn genotypes ranged from 233 to 588 mg/kg, 176 to 440 mg/kg, and 216 to 617 mg/kg at the Cambridge, Huxley, and South Amana sites, respectively. It is known that the opaque-2 mutation in corn (high lysine genotypes) is associated with an increased level of free amino acids in grains. According to the results of Wang and Larkins,^34^ the concentration of Asn in native corn was one-ninth of this opaque-2 mutant. Analyzing the free amino acid in cereals, Kocadağlı et al.^35^ found that the free Asn content was lower in different colored corn grains compared to that in wheat and hulless barley. The content was 224 and 275 mg/kg in standard seeded yellow and blue corn, respectively, and 267 and 268 mg/kg in dark-blue and dark-red popping corn, respectively, grown in the 2014 season at the Maize Research Institute, Serbia. However, Žilić et al.^36^ reported that the content of free Asn in the grain of the same genotype of dark-blue popping corn grown at the same location in the 2018 season was higher by about 31%. The same authors determined a high variation in the free Asn content between the examined corn genotypes, from 190 mg/kg in standard seeded red corn to 471 mg/kg in standard seeded yellow corn. According to Žilić et al.^37^ the coefficient of variation for free Asn in corn genotypes grown in the field at the Maize Research Institute, Serbia, in the 2015 growing season was 28.2%. The concentration of free Asn ranged from 355 to 649 mg/kg. The free Asn concentration of the grain responds to the nutrient availability in the soil. Sulfur deficiency in the soil can cause an increase in the accumulation of free Asn in corn grains. Free Asn was predominant in total free amino acids in sulfur starved corn hybrid grains of the INRA (National Institute of Agricultural Research in France), and the amount of Asn was reported to be 10-fold of the control.^38^ Nitrogen fertilization has the opposite effect. However, according to the early work of Shewry et al.^39^ the ratio of sulfur:nitrogen was an important determinant of the asparagine accumulation rather than nitrogen alone. Under soil sulfur-deficient conditions, the synthesis of prolamine in the grain was reduced, while the content of free amino acids, as well as the proportion of asparagine in the total amino acid content, was increased.^39^ Further, Asn can become the predominant free amino acid in cereal grains under stress conditions.^40^ The impact of water restriction on the levels of free amino acids in corn highlighted extensive hybrid dependence.^41^ By comparing seven tested hybrids, the authors showed a significant interaction between genotypes and dry treatments (*p* < 0.05) for the amount of free Asn. The free Asn content ranged from 189 to 637 mg/kg (on average 325 mg/kg), 188 to 568 mg/kg (on average 315 mg/kg), and 143 to 341 mg/kg (on average 232 mg/kg) in corn genotypes grown under the water-restricted conditions during grain filling, water-restricted conditions during the vegetative phase, and well-watered regime, respectively.^41^ Anjum et al.^42^ also found that drought stress triggered the accumulation of total free amino acids in tested corn hybrids. Accumulations of free amino acids increased with the severity of drought stress (severe drought > moderate drought > low drought > control; irrigation levels of 40, 60, 80, and 100% of field capacity, respectively).^42^ According to a study by Navari-Izzo et al.^43^ total protein amino acid levels in corn when water was in deficit dropped by 40% while the free amino acid content increased by 2.5-fold. Further, Canas et al.^44^ found that free Asn was predominant in the “aborted grains” in all examined corn genotypes and its contribution ranged from 24 to 44% of the total free amino acids. There is evidence that the plants exposed to toxic metals, pathogen attack, and salt stress accumulate Asn especially in the vegetative parts.^40^ However, there are few studies on the effect of these stress conditions on the free Asn content in corn grains in general.

Summarizing the above results, it can be concluded that, depending on genetic factors and environmental conditions, the content of free Asn in corn varies in a wide range from 140 to 650 mg/kg.

### Reducing Sugars

Based on the summarized results by Halford et al.,^45^ the maximum amounts of glucose (Glc), fructose (Fru), and sucrose (Suc) in the grains of corn, although affected by environmental and genetic factors, were higher by 30, 45, and 71%, respectively, than that in wheat grains. However, the maximum amounts of Glc and Fru were just over two-thirds of their content in the grains of rye. Žilić et al.^37^ reported that Suc was the most abundant sugar in the grains of dent corn (*Zea mays* var. *indentata*). In investigated corn genotypes the content ranged from 0.70 to 1.75% of dry matter. Even though Suc is nonreducing, it can contribute to acrylamide formation either through its hydrolysis into Glc and Fru or through its degradation to Glc and a very reactive fructofuranosyl cation.^46^ Žilić et al.^37^ also reported that Glc with a content of 0.16–0.53% was the predominant reducing monosaccharide in corn grains, while maltose (Mal) with a content of 0.35–0.72% was the predominant reducing disaccharide. The Fru content varied from 0.09 to 0.35%. Table 1 shows the content of sugars in the grain of flint corn (*Zea mays* var. *indurate*), popping corn (*Zea mays* var. *everta*), and sweet corn (*Zea mays* convar. *saccharata* var. *rugosa*) grown at the same location in Serbia during the 2017 growing season. The highest total sugar content was determined in sweet corn varieties. The sugar content in these genotypes ranged from 3.60 to 8.72% of dry matter with a maximum content of Glc, Fru, Suc, and Mal of 2.87, 1.55, 3.70, and 0.57%, respectively, in the grain of the white-colored genotype. As shown in Table 1, the content of total reducing sugars in the tested corn genotypes ranged from 0.52 to 5.02%. The percentage contribution of reducing monosaccharides and reducing disaccharides to the total sugars’ content varied from about 13 to 51% and from 2 to 54%, respectively. The effects of genetic and environmental factors on the content of all detected sugars were confirmed by high coefficients of variation (Table 1). Otherwise, a different distribution of monosaccharides and disaccharides within corn grain was observed. In normal corn grain, the highest sugar levels were detected in the base and pericarp with particularly high Glc and Fru contents in the basal region. A Suc content gradient appeared to exist between the basal region and the upper endosperm of normal corn grain.^47^ Like the asparagine content, the sugar content in corn grain is also subject to natural variations to some extent. According to the early research of Kereliuk et al.^48^ the amounts of Glc, Fru, and Suc in corn hybrids grown at three locations in North America ranged from 0.18 to 0.32%, 0.14 to 0.32%, and 2.79 to 3.60%, respectively. By analyzing the corn hybrids developed by crossing of 48 inbred lines to two different testers/parents and grown at three locations in the United States, an effect of a location on the amount of Suc was established.^33^ Effects of a noninteracting tester and location were observed for Glc and Fru.^33^ Harrigan et al.^41^ set up an experiment with corn hybrids grown in different water regimes (water-restricted conditions during grain filling, water-restricted conditions during the vegetative phase, and well-watered regime). Of the sugars tested, glucose showed a treatment effect when calculated across all corn hybrids.^41^ The Glc amount was generally highest in the corn exposed to conditions of the water-restricted regime during the vegetative phase and lowest in those exposed to conditions of the water-restricted regime during grain filling.^41^ According to the study of Kawatra et al.^49^ under conditions of limited irrigation, the reducing sugar content of corn grains decreased by 1.5- to 1.9-fold at the different stages of the grain development (7, 14, 21, 28, and 35 days of initiation of silking) over those of control samples, while an increase in the sucrose content was observed. Data presented by Jood et al.^50^ indicated a significant decrease in the content of total, reducing, and nonreducing sugars in corn grains infested by two insect species (*Trogoderma granarium* Everts and *Rhizopertha dominica* Fabricius) individually and in the mixture. With the increase in the level of infestation, there was a progressive increase in the loss of sugars. The content of total, reducing, and nonreducing sugars in the control/uninfected corn sample was 4.71, 1.80, and 2.91%, respectively. *R. dominica* caused significantly higher losses of total sugars (5–49%), reducing sugars (4–40%), and nonreducing sugars (5–55%) at 25, 50, and 70% of corn grains’ infestation, respectively, as compared to *T. granariurn* showing 1–15%, 1–12%, and 1–17% losses, respectively. The sugar content of corn grains may also be correlated with the storage conditions. For example, Jood et al.^50^ determined the increase of the content of total, reducing, and nonreducing sugars ranging from 2 to 17% in corn grains as a consequence of starch breakdown after the four months’ storage at 29–39 °C and a humidity of 60–90%.

**Table 1:** Content of Sugars (% of d.m.) in Different Corn Genotypes^a^

	**Glc**	**Gal**	**Fru**	**Suc**	**Mal**	**TS**	**TRS**	**IRS**	**IRmS**	**IRdS**
*Zea mays* var. *indurate* – (flint corn)										
White grain	0.58	n.d.	0.46	0.56	0.55	2.16	1.60	74.2	48.6	25.6
Yellow grain	0.36	n.d.	0.13	0.50	0.53	1.53	1.03	67.5	33.0	34.4
Blue grain	0.33	0.02	0.08	1.55	1.25	3.24	1.69	52.3	13.5	38.8
Red grain	0.40	n.d.	0.11	0.66	1.01	2.19	1.53	69.9	23.7	46.3
**CV**	26.8		91.2	60.3	42.5	31.1	20.2	14.5	50.3	23.8
*Zea mays* var. *everta* – (popping corn)										
White grain	0.28	n.d.	0.15	0.90	0.89	2.22	1.32	59.4	19.2	40.2
Yellow grain	0.26	n.d.	0.08	0.42	0.92	1.70	1.27	75.0	20.7	54.2
Blue grain	0.21	n.d.	0.13	0.72	0.48	1.55	0.82	53.0	21.9	31.1
Red grain	0.14	n.d.	0.12	0.67	0.24	1.19	0.52	43.3	22.6	20.7
**CV**	28.0		24.5	29.2	52.1	25.7	38.8	23.1	7.0	38.9
*Zea mays* convar. *saccharata* var. *rugosa* – (sweet corn)										
White grain	2.89	n.d.	1.56	3.70	0.57	8.72	5.02	57.5	51.0	6.53
Yellow grain	1.12	n.d.	0.21	1.88	0.19	3.60	1.72	47.9	42.6	5.32
Blue grain	1.16	n.d.	0.44	2.04	0.50	4.15	2.10	50.7	38.6	12.1
Red grain	1.19	n.d.	0.41	2.77	0.09	4.46	1.69	37.9	35.9	1.98
**CV**	54.5		93.4	32.0	69.2	44.9	60.9	16.8	15.7	65.1

a n.d., not detected; TS, total sugars; TRS, total reducing sugars; IRS, index of reducing sugars (percentage of reducing sugars in the content of the total); IRmS, index of reducing monosaccharides (percentage of reducing monosaccharides in the content of the total); IRdS, index of reducing disaccharides (percentage of reducing disaccharides in the content of the total).

## Occurrence of Acrylamide in Corn-Based Food Products

Corn-based products constitute one of the major components of the human diet in many cultures, and hence, the risk of acrylamide formation has a significant impact on human health. In this regard, there are scientific papers presenting the acrylamide content in different corn-based food products. The results presented in 48 scientific papers are summarized in Table 2. An overview of the acrylamide content in corn-based food products prepared in laboratories, collected from industry and purchased from supermarkets, local stores, and restaurants worldwide is provided. According to the results of numerous authors (Table 2), the acrylamide content in corn/tortilla chips, popcorn, and corn flakes, as widely consumed products all over the world, ranged from 5 to 6360 μg/kg, < LOQ to 2220 μg/kg, and from not detected to 1186 μg/kg, respectively. The acrylamide content in corn-based biscuits and corn-based snack products bought mainly in the European market ranged from < LOQ to 325 μg/kg and from <5 to 923 μg/kg, respectively (Table 2). The content of acrylamide in gluten-free corn-based products was lower than in standard commercial products and ranged from 5.7 μg/kg in yellow corn flatbread to about 65 μg/kg in infant cereal formulation (Table 2).

**Table 2:** Acrylamide Content in Corn-Based Processed Foods^a^

				**Acrylamide (μg/kg)**			
**Year of research**	**No. of samples**	**Reference/Country**	**Subject of research**	**min**	**max**	**mean**	**Food products**
2002	16	Smiciklas-Wright et al.^98^ (U.S.A.)	Acrylamide levels in foods commonly consumed in the United States.	111	240	199	Corn/tortilla chips
	15			97	352	180	Popcorn
	16			n.d	15	6	Tortillas
2003	3	Svensson et al.^63^ (Sweden)	Industrially produced foods available on the Swedish market analyzed for acrylamide.	120	180	150	Tortilla crisps
	3			365	715	500	Popcorn
2003	12	Konings et al.^99^ (Netherlands)	Acrylamide exposure from foods to the Dutch population.	<30	300	121	Corn flakes
2003	5	Leung et al.^100^ (China)	Acrylamide content of Asian foods available in Hong Kong prepared in Chinese, Japanese, Indian, Indonesian, Malaysian, Thai, and Vietnamese styles. (Some Western foods were also included for comparison purposes.)	65	230	300	Corn-based crisps
	1						Corn flakes
2003	6	Jung et al.^88^ (South Korea)	Effect of lowering pH on acrylamide formation in fried and baked corn chips (0.1 and 0.2% citric acid, frying with corn oil at 180 °C for 30 s, baking at 255 °C for 100 s).	≈23	≈152		Corn chips
2004	15	Murkovic^101^ (Austria)	Ready-to-eat products from the Austrian market analyzed for acrylamide.			106	Popcorn
2004		Hilbig et al.^102^ (Germany)	Estimation of the dietary intake of acrylamide by German infants, children, and adolescents as calculated from available data on acrylamide levels in food groups (BVL data).	n.d.	846		Corn flakes
2005	5	Matthys et al.^60^ (Belgium)	Dietary acrylamide intake in Flemish adolescents (food items collected from different supermarkets and restaurants).	129	216		Popcorn
2006	16	Rufián-Henares et al.^103^ (Spain)	The relationship among levels of acrylamide and the compositional parameters of the samples (Breakfast cereals randomly purchased in different supermarkets in 2006).			207 ± 55	Corn-based breakfast cereals
2007	2	Eerola et al.^104^ (Filand)	Acrylamide levels in Finnish foodstuffs (Samples purchased in local retail shops and fast-food restaurants).	180	210	195	Corn snacks
	3			260	350	300	Popcorn
2007	8	Arisseto et al.^105^ (Brazil)	Acrylamide levels in selected foods in Brazil.	<LOQ	49		Corn-based breakfast cereal
	3			<LOQ	33		Deep-fried polenta
2008	110	Mills et al.^106^ (U.K.)	Dietary acrylamide exposure estimates for the United Kingdom.	10	545	98	Corn-based cereal products
	42			40	820	201	Corn-based snacks
2008	9	Ölmez et al.^107^ (Turkey)	A survey of acrylamide levels in foods from the Turkish market.	109	835	429	Corn chips
	7			35	478	122	Corn flakes
	1					171	Popcorn
	3			100	288	194	Roasted corn
2010	24	Mojska et al.^58^ (Poland)	Acrylamide content in Polish foods (Foodstuffs taken at randomly selected stores and catering establishments all over Poland).	70	1186	223	Corn flakes
	3			124	300	188	Corn chips
2010	2	Boroushaki et al.^108^ (Iran)	Acrylamide in popular Iranian brands’ corn products from the domestic food industry (Seven brands of corn products collected from a factory before packaging and 1 brand collected from a market. Popcorn, 200–220 °C for 2 min; cheese covered snack, 80–120 °C for less than one second in extruder; fried snack after extruding, 160 and 165 °C for 4 min).	≈380	≈400		Popcorn
	4			≈30	≈60		Corn-based snack (cheese covered)
	2			≈30	≈100		Corn-based fried snack
2012		Cressey et al.^109^ (New Zeland)	Acrylamide in New Zealand foods (Samples purchased from retail outlets).	410	734	596	Corn chips
				81	228	154	Popcorn
						110	Corn flakes
2012	9	Sun et al.^27^ (China)	Acrylamide content in microwaved and conventionally heated popcorn (Prepared in a laboratory for 4 min in a 1000 W microwave oven; different flavors added).	≈166	≈2220		Popcorn
2012	22	Cheng et al.^110^ (Taiwan)	Acrylamide content of snack foods surveyed in Taiwan (Snack food samples purchased in supermarkets in Taipei).	<5	403	271	Corn-based snack
2013	12	Normandin et al.^56^ (Canada)	The distribution of acrylamide in food items frequently consumed by Canadian adolescents (Canadian Urban Center).	265	384	325	Corn chips
	4			213	457	329	Popcorn
2013	4	Žilić et al.^24^ (Serbia)	Effects of infrared heating on Maillard reaction products in corn flakes (Infrared heating performed using a micronizer. Corn grains heated for 50–100 s and the output set to 110, 115, 120, and 140 °C).	159 ± 5	705 ± 26		Corn flakes
2014	8	Salazar et al.^5^ (Mexico)	Effect of added calcium hydroxide during corn nixtamalization on the acrylamide content in tortilla chips (Lab scale production with Ca(OH)_2_ at concentrations of 0.5, 1.0, 1.5, and 2.0 g/100 g corn, fried in soybean oil at 180 °C for 30 and 45 s).	≈346	≈1066		Tortilla chips
2014	9	Delgado et al.^111^ (Mexico)	Effect of water activity on the acrylamide content in tortillas (Lab scale).	≈696	≈1344		Tortilla chips
2015	23	Hariri et al.^112^ (Lebanon)	Quantification of acrylamide in baked and fried corn chips (Local and imported brands of chips randomly collected from various locations across Lebanon).	329	6360	1574	Corn chips
2015	5	Pacetti et al.^113^ (Columbia)	Acrylamide levels in selected Colombian foods.	<LOQ	781	452	Popcorn
	9			78	441	253	Corn chips, corn nut
	3			<LOQ		<LOQ	Arepa (corn patty)
2015	2	Capei et al.^114^ (Italy)	Acrylamide levels in biscuits and breakfast cereals (Samples of the most consumed brands in Italy (17 and 5, respectively) randomly collected in the two major supermarkets in Florence).	<LOD	30		Corn/wheat-based biscuits
	1					280	Corn/barley-based biscuits
	1					360	Corn/rice-based breakfast cereals
	1					<LOD	Corn/barley-based breakfast cereals
	1					110	Corn/oat/rice/cocoa-based breakfast cereals
2016	8	Makowska et al.^93^ (The Czech Republic)	Acrylamide contents in corn snacks containing 0, 3, 5, and 10% of nanofiltered whey powder, obtained from the raw material of 12 and 14% moisture contents after extrusion.	291	887		Corn-based snacks
2016	204	Claeys et al.^115^ (Belgium)	Acrylamide in different foodstuffs purchased in the Belgian market in the period of 2002–2013.	<LOQ	1100	220	Popcorn
2016	77	Alyousef et al.^116^ (Syria)	Acrylamide levels in different brands of commercial and traditional foodstuffs available in Syria (Food products purchased in different local supermarkets).	57 ± 3	325 ± 2		Corn biscuits, wafers, crackers
	6			183 ± 3	366 ± 5		Corn chips
2016	10	Delgado et al.^70^ (Mexico)	Acrylamide content in tortilla chips prepared from pigmented corn grains (Lab scale production with 1 g Ca(OH)_2_/100 g corn, fried in soybean oil at 180 °C for 30 and 45 s).	≈85	≈1660		Tortilla chips
2017	20	Esposito et al.^65^ (Italy)	Acrylamide levels in potato crisps and other snacks (Samples of ten different brands bought in local stores).			257 ± 122	Corn-based extruded snacks
2017	14	Hu et al.^117^ (China)	Acrylamide in thermal-processed carbohydrate-rich foods from the Chinese market.	18	1966	524 ± 187	Corn products, including cornflakes and popcorn
2017		Sánchez-Otero et al.^57^ (Mexico)	Estimation of the acrylamide content in foods consumed by young people in Mexico and calculation of its intake in this population (Commercial starchy foodstuffs selected in local supermarkets, fast-food restaurants, and convenience stores).			498 ± 19	Corn breakfast cereal
2018	14	Juodeikiene et al.^94^ (Lithuania)	Effect of infrared and microwave heating on acrylamide formation (Corn flour was heated for 10, 7.5, and 5 min at 60 °C by microwave and infrared waves for 10 s at 76 and 90 °C).	≈10	≈120		Thermally treaded corn flour fraction
2019	4	Topete-Betancourt et al.^90^ (Mexico)	Effect of different processes on mitigation of acrylamide formation in tortilla chips (Classic-ash, traditional-lime, ecological-carbonate, and extrusion-only water).	46 ± 1	1443 ± 4		Tortilla chips
2019	61	Abt et al.^52^ (U.S.A.)	Acrylamide level and dietary exposure from foods in the United States (Samples from retail markets or restaurants throughout the United States).	5	610	220	Tortilla chips
2019		Mesías et al.^61^ (Spain)	Influence of the predominant cereal, the presence of honey, and the manufacturing process on the acrylamide levels (Cereal products made by more than 20 producers purchased in different supermarkets in 2018).			≈70	Corn-based breakfast cereals
2019	4	Mesías et al.^62^ (Spain)	Acrylamide content in the Spanish biscuit market (Commercial biscuits made by 30 different producers purchased in different supermarkets. Biscuits containing dried fruits, nuts, chocolate, or jam).	≈20	≈250	≈50	Corn-based biscuits
2019		Crawford et al.^118^ (U.S.A.)	Acrylamide level in 15 experimental flatbreads made from gluten-free cereals and 21 standard commercial flatbreads.			5.7 ± 2.2	Gluten-free flatbread based on organic yellow corn
						8.8 ± 1.1	Gluten-free flatbread based on enriched and degermed corn
2017		Sánchez-Otero et al.^57^ (Mexico)	Estimation of the acrylamide content in foods consumed by young people in Mexico and calculation of its intake in this population (Commercial starchy foodstuffs selected in local supermarkets, fast-food restaurants, and convenience stores).			498 ± 19	Corn breakfast cereal
2018	14	Juodeikiene et al.^94^ (Lithuania)	Effect of infrared and microwave heating on acrylamide formation (Corn flour was heated for 10, 7.5, and 5 min at 60 °C by microwave and infrared waves for 10 s at 76 and 90 °C).	≈10	≈120		Thermally treaded corn flour fraction
2019	4	Topete-Betancourt et al.^90^ (Mexico)	Effect of different processes on mitigation of acrylamide formation in tortilla chips (Classic-ash, traditional-lime, ecological-carbonate, and extrusion-only water).	46 ± 1	1443 ± 4		Tortilla chips
2019	61	Abt et al.^52^ (U.S.A.)	Acrylamide level and dietary exposure from foods in the United States (Samples from retail markets or restaurants throughout the United States).	5	610	220	Tortilla chips
2019		Mesías et al.^61^ (Spain)	Influence of the predominant cereal, the presence of honey, and the manufacturing process on the acrylamide levels (Cereal products made by more than 20 producers purchased in different supermarkets in 2018).			≈70	Corn-based breakfast cereals
2019	4	Mesías et al.^62^ (Spain)	Acrylamide content in the Spanish biscuit market (Commercial biscuits made by 30 different producers purchased in different supermarkets. Biscuits containing dried fruits, nuts, chocolate, or jam).	≈20	≈250	≈50	Corn-based biscuits
2019		Crawford et al.^118^ (U.S.A.)	Acrylamide level in 15 experimental flatbreads made from gluten-free cereals and 21 standard commercial flatbreads.			5.7 ± 2.2	Gluten-free flatbread based on organic yellow corn
						8.8 ± 1.1	Gluten-free flatbread based on enriched and degermed corn
2020	10	Merhi et al.^54^ (Lebanon)	Determination of carcinogenic and neurotoxic risks associated with acrylamide intake from cereal products (Cereal products, both local and imported, randomly collected from several locations in Lebanon).	141	373	220	Corn-based cereal products
2020	8	Bušová et al.^119^ (The Czech Republic)	Acrylamide levels in different foods available in the Czech Republic market.	433	1410	761 ± 304	Popcorn
	9			50	191	115 ± 42	Corn flakes
2020	6	Mandić Andačić et al.^66^ (Croatia)	Arylamide in different types of bread and bakery products before and after European regulation of acrylamide reduction (Samples of bread and bakery products from different parts of Republic of Croatia collected between 2015 and 2018 and in 2018).	<LOQ	82	56 ± 13	Corn-based bakery products
	2			<LOQ	34	27 ± 10	Corn-based bakery products
2020	4	Žilić et al.^36^ (Serbia)	Acrylamide formation in biscuits made of different whole-grain flours (Biscuits prepared in the laboratory from 100% flour of white-, yellow-, blue-, and red-colored corn and baked for 7, 10, and 13 min at 180 °C).	24–69	95–321		Corn biscuits
2021	5	FDA^120^ (U.S.A.)	Acrylamide level in foods in the United States market.	54	77		Corn flakes
	4			164	240		Tortilla chips
	4					n.d.	Corn bread-homemade
	4					n.d.	Corn/hominy grits
	4			97	352		Popcorn
2021	3	Kamankesh et al.^121^ (Iran)	Optimization of the acrylamide extraction method and investigation of the composition, temperature, and heating time in the acrylamide formation in snacks including corn-based snacks.	162	259	218 ± 50	Popcorn
	9			116	923	369 ± 350	Extruded corn snack
	2			226	447	337 ± 157	Corn snack
2021	1	Verardo et al.^29^ (Spain)	Influence of gluten-free and gluten-rich cereals’ formulation on the acrylamide content in infant food.			≈65	Gluten-free infant formulation based on corn and rice
	5			≈80	≈95		Infant formulation with added corn

a LOD, limit of detection; LOQ, limit of quantitation.

## Human Exposure to Acrylamide from Corn-Based Food Products

More than one-third of food products consumed by the U.S. and European populations contain acrylamide. Therefore, concern for public health implies, among other things, an assessment of whether the intake of acrylamide at levels found in the food supply is an important health risk factor. According to a risk assessment made by the European Food Safety Authority,^51^ mean and 95th percentile dietary acrylamide exposures across all European age groups were estimated as 0.4–1.9 μg/kg of body weight (bw) per day and 0.6–3.4 μg/kg of body weight per day, respectively, with the highest intake in adolescents and children. For the U.S. population over 2 years of age, the estimated mean dietary acrylamide exposure was 0.36 μg/kg of bw per day (at 90th percentile: 0.86 μg/kg of bw per day) and 1.42 μg/kg of bw per day (at 90th percentile: 3.02 μg/kg of bw per day) for those below 2 years of age.^52^ The tolerable daily intake and margins of exposure for neurotoxicity from acrylamide for an average consumer were estimated to be 40 μg/kg per day and 300, respectively. For cancer, they were 2.6 μg/kg per day and 200, respectively.^53^ However, according to Eisenbrand^2^ at single dosages up to at least 100 μg/kg bw (which strongly exceeds present-day average consumer exposure), DNA damage was not found to be dose-related and remained at the lower bound of human background DNA damage of comparable DNA N7-Gua lesions.

Cereal foods significantly contribute to acrylamide intake. However, dietary preferences among different countries affect the total contribution of cereal products and the importance of different food categories within the cereal group. For example, according to the data from a recent study by Merhi et al.,^54^ the dietary exposure of the Lebanese population (from the age of 3 to the age of 75) to acrylamide from the various types of cereals was found to be 0.9 μg/kg of bw per day (corn), 1 μg/kg of bw per day (wheat), 0.7 μg/kg of bw per day (rice), and 0.7 μg/kg of bw per day (oat). While the acrylamide margin of exposure from corn-based food products does not appear to pose a health concern for the entire Lebanese population, children and teens are subjected to a high chronic carcinogenic risk with margin of exposure values well below 100. According to the calculation of the OEHHA,^55^ the daily intake of acrylamide from tortillas (corn or flour), corn flakes, popcorn, and corn chips/tortilla chips in the United States population was 0.04–0.41 μg/day/capita, 1.41–3.46 μg/day/capita, 0.47–4.32 μg/day/capita, and 0.80–9.15 μg/day/capita, respectively. The acrylamide intake of 1 μg/day/capita would be exceeded if one corn chips and popcorn unit (the amount of a given food that is consumed on average per day with the average acrylamide amount in it) were consumed on average once every 9 and 4 days, respectively. For comparison, the same value would be exceeded if one French-fried potato unit was consumed once every 26 days. Among the 20 foods, Abt et al.^52^ ranked corn snacks 12th by acrylamide intake in the U.S. population over 2 years of age for the 2002–2006 period with a mean value of 0.011 μg/kg of bw per day. However, the results of the 2011–2015 data indicate that corn snacks have climbed to sixth place on the list of top foods contributing to acrylamide exposure.^52^ The average contribution of corn chips and popcorn to the total acrylamide intake among adolescents in Canada was 5 and 4%, respectively, i.e. on average 0.03 μg/kg of bw per day.^56^ In the Mexican population of average age of 22 years, the exposure to acrylamide from corn breakfast cereal was 2.18 ± 7.78 μg/kg product for 19.71% of surveyed subjects. The exposure to acrylamide from microwave popcorn was lower and amounted to 0.92 ± 4.3 μg/kg product for 6.57% of surveyed individuals. Based on these data, a daily intake of about 0.031 ± 0.11 and 0.013 ± 0.06 μg/kg of bw was estimated.^57^ In the Polish young population, a significant intake of acrylamide also originates from corn-based food products. Corn flakes and corn crisps supplied altogether 5% of acrylamide in the group of children and adolescents aged 7–18 years and 10% in the group of children aged between 1 and 6 years.^58^ In a population of Polish girls and boys from an urban environment, the 95th percentile dietary intake of acrylamide by corn flakes’ consumption was 0.09 and 0.14 μg/kg of bw per day, respectively.^59^ In boys’ diets, corn flakes were a more significant contributor of acrylamide compared to French fries and salty sticks.^59^ The mean consumption of popcorn in Flemish adolescents was 0.14 g/day. According to research, girls consumed three times more popcorn than boys.^60^ Compared to the contribution of wheat-, oat-, rye-, spelt-, barley-, rice-, and quinoa-based breakfast food to the daily acrylamide exposure of the Spanish population, the consumption of corn-based breakfast food caused intermediate exposure (values ranged from 0.12 to 0.72 μg/day/capita).^61^ Since biscuits are an important acrylamide source in the common diet of all age populations, a calculation of the acrylamide exposure from this food category for the Spanish population was done by the same authors.^62^ The daily exposure to acrylamide from corn-based biscuits was 1.91 ± 2.44 μg/day/capita. In 2013, the Danish National Food Institute published the food categories mostly contributing to the intake of acrylamide in the children’s population. Potato products ranked first and were followed by corn crisps.^51^ In addition, potato crisps and popcorn contributed most to the acrylamide intake in young adults aged 18–34 years in Sweden. The dietary intake of acrylamide in the Swedish population (age 18–74 years) from tortilla crisps and popcorn consumption ranged from 0 to 2.4 μg/person/day and from 0 to 57 μg/person/day, respectively.^63^ The contribution of the snacks (peanuts and popcorn) to the dietary exposure to acrylamide in pregnant Norwegian women was 7 to 12% depending on the applied calculation method.^64^ According to the study of Esposito et al.,^65^ the mean and 95th percentile dietary acrylamide exposures by corn-based extruded snacks (corn curls and corn chips) consumption ranged from 0 to 0.08 μg/kg of bw per day and 0 to 0.775 μg/kg of bw per day, respectively, across all Italian age groups. Higher intake values occurred among younger generations (toddlers, other children, and adolescents). For these age groups, the maximum values of the 95th percentile acrylamide intake through corn-based extruded snacks were 0.775, 0.41, and 0.121 μg/kg of bw per day, respectively. Mandić Andačić et al.^66^ estimated the mean exposure to acrylamide of the Croatian adult population through the consumption of four groups of bread and bakery products. The dietary intake of acrylamide from a corn-based group of products in this population was 0.056 μg/kg of bw per day. The range of contributions of cereals to the total acrylamide intake among all people of all ages in China was 26.1–34.2%.^67^ However, corn-based food products, as a separate group of contributors to their acrylamide exposure, are infrequently presented in the scientific papers.

## Benchmark Levels of Acrylamide

Since 2005 the EFSA has recognized the presence of acrylamide in food. Shortly thereafter, the European Commission issued Commission Recommendation 2007/331/EC^68^ on monitoring the level of acrylamide in food. Based on the EFSA data monitored in the 2007–2012 period, in 2013, the European Commission published Recommendation 2013/647/EC^69^ regarding the analysis of acrylamide levels in foods, in which the indicative values for acrylamide are presented. In 2017, the European Commission published Regulation 2017/2158/EC, establishing mitigation measures to reduce the presence of acrylamide in food and its benchmark levels in some food categories.^69,7^ Apart from the EU Member States, which are obliged to comply with the Regulation, many other countries worldwide have adopted these benchmark values for the acrylamide content in certain foods. Although the dietary exposure to acrylamide has been identified as a potential concern, there are no set levels for acrylamide for food that is sold, for example, in Canada, Australia, New Zealand, and Turkey. Corn-based food products are not specifically categorized by the European Commission Regulations. Naturally, these products are classified in the group of cereal-based food products. Therefore, the recommended level of acrylamide in them should be lower than the prescribed benchmark levels in different categories of cereal-based food products. Benchmark levels for cereal-based food products, as defined in Regulation of European Commission 2017/2158/EC,^7^ are 300 μg/kg for whole-grain-based breakfast cereals and 150 μg/kg for non-whole-grain-based breakfast cereals, 100 μg/kg for soft bread other than wheat-based bread, 350 μg/kg for biscuits and wafers, 400 μg/kg for crackers with the exception of potato-based crackers, 150 μg/kg for biscuits and rusks for infants and young children, and 40 μg/kg for baby foods, processed cereal-based foods for infants and young children excluding biscuits and rusks.

It seems practical to use the acrylamide benchmark levels of other cereal-based food products for corn-based products for now because of their similar free asparagine content and the similarity of the processes used for their production. However, it is considerable that the benchmark levels of other cereal products will not be applicable to the widely consumed corn-based products such as tortilla chips and popcorn. Therefore, monitoring of the acrylamide levels of corn-based products before and after application of reduction strategies for a yearly based period could be the starting point for regulators to determine the lowest applicable acrylamide levels by the industry.

## Acrylamide Mitigation Strategies in Corn-Based Food Products

In addition to the European Commission, renowned food organizations such as the U.S. Food and Drug Administration, the Codex Alimentarius Commission, and the FoodDrinkEurope Toolbox have published several documents that provide guidance for acrylamide mitigation. In general, the strategies recommended for reducing acrylamide in food, and thus in corn-based food, can be categorized into five different groups:

### Effect of Raw Materials

Selecting corn genotypes with a low content of reducing sugars and primarily with a low content of free asparagine may help reduce acrylamide while maintaining the desired product qualities.^33,36,37^ Additionally, certain natural compounds present in corn, depending on the genotype, can affect the acrylamide formation in corn-based thermally treated foods. An example of this is the study carried out by Delgado et al.^70^ in which authors suggested that selected corn genotypes rich in anthocyanins and with lower levels of fat and phenolic compounds could reduce the acrylamide formation in tortilla chips. According to the results of Žilić et al.,^36^ a lower content of acrylamide was determined in biscuits prepared from anthocyanin-rich whole-grain flour of red- and blue-colored corn and baked at 180 °C for 7, 10, and 13 min than in white corn- and yellow corn-based biscuits. After 13 min of baking, the acrylamide content in the red corn-based biscuits was lower by about 70 and 60% than that in the biscuits made from the flour of two anthocyanin-free corn genotypes, respectively. However, up to now, there has been no report describing the mechanistic role of anthocyanins on acrylamide formation in foods. Only a few studies reported the inhibition of acrylamide toxicity by anthocyanins in both cell and animal models, and this was mostly attributed to the prevention of acrylamide-induced oxidative stress.^71,72^

### Effect of Crop Management Regimes

Sulfur fertilizers and the well-watered regime, i.e. irrigation practice, reduce the content of acrylamide precursors in corn grain, i.e. free asparagine, while nitrogen fertilizers have the opposite effect.^32,38,41^ Nitrogen fertilizer application was found to increase asparagine levels in different crops owing to upregulation of asparagine synthetase gene expression.^73,74^ Claus et al.^73^ reported that nitrogen fertilization significantly increased the free asparagine concentration to 220.3 mg/kg from 54.0 mg/kg by application of 200 kg of N/ha in a wheat variety (Enorm). A similar result was provided by Weber et al.^74^ Application of 180 kg N/ha caused a 3.5 times increase in free asparagine compared to untreated controls in winter wheat (*Triticum aestivum* L.). On the other hand, asparagine in wheat grain is affected by sulfur application more than cysteine and methionine, although it does not contain sulfur.^75^ High amounts of free asparagine were found in wheat flours which were grown in limited sulfur, whereas much lower amounts were obtained from flours grown at saturated conditions.^76^ Sulfur-deficient barley was reported to contain a reduced amount of total protein and increased content of nonprotein amino acids.^77^ In this case, nonprotein amino acids in barley contained increased aspartic acid + asparagine content. Similarly, sulfur deficiency in maize kernels was reported to give rise to an increase in free asparagine.^78^ Considering increased free asparagine content under limited sulfur conditions, it is suggested to eliminate sulfur deficiency in crops in terms of acrylamide mitigation. Application of sulfur at a rate of 50 kg sulfur per hectare is recommended by UK’s Agriculture and Horticulture Development Board to keep the free asparagine concentration as low as possible in wheat to minimize acrylamide formation.^79^ In addition to this, in Sweden, application of sulfur fertilizers accompanied by nitrogen is followed as “good agricultural practices”, and this is also implicated as a compulsory mitigation strategy in European Commission Regulation (EU) 2017/2158 (EC, 2017).^7^ Besides fertilization, infection of crops by pathogens also affects the asparagine concentration in many crops. The studies in wheat grains to date indicate that the lack of fungicide treatment results in accumulation of asparagine.^80,81^ Accordingly, effective disease control is one of the crop management strategies for acrylamide mitigation, and thus, prevention of fungal infection is considered as another application of good practice on crop protection by European Commission Regulation (EU) 2017/2158 (EC, 2017).^7^ Similar protection measures should also be applied in corn production in order to control the acrylamide formation in corn-based snacks. In addition, postharvest control, i.e. control of corn grain storage conditions, can be accepted as a possible strategy to reduce acrylamide formation. In order to slow down deterioration processes, corn grains should be protected from moisture and temperature, the growth of microorganisms, and pest attacks during storage. The moisture content of grains below 11% and the storage room temperature below 20 °C and 50% humidity are desirable conditions for a longer period of corn storage.^82^

### Effect of Additives

The addition of antioxidants, asparaginase, amino acids such as lysine and glycine, and salts (Na^+^, Mg^2+^, or Ca^2+^) before heat processing of foods has been proposed as a possible strategy to reduce acrylamide formation.^83^ For example, the use of MgCl_2_ as a divalent cation in masa preparation was reported as an effective mitigation strategy in tortilla chips with a reduction of acrylamide by 69–74% depending on the concentration of the salt used. Similarly, the use of CaCl_2_ in masa preparation reduced acrylamide by 52% to 67% in tortilla chips.^84^ Adding asparaginase to masa reduced the acrylamide in tortilla chips by 90%.^85^ Another study showed that the addition of the amaranth protein isolate to the recipe of masa decreased the acrylamide content by 51% and 62% in fried tortilla chips and baked tortilla chips, respectively.^86^ The decrease was explained not only by the fact that the amino acids in the amaranth protein isolate competed with asparagine to react with carbonyl compounds but also by the fact that the remaining amino acids could react with the formed acrylamide. The reaction of acrylamide with amines, amino acids, and polypeptides was studied by Zamora et al.^87^ to explain the fate of acrylamide during storage and after heating. According to that study, Michael addition of amino compounds to acrylamide forms 3-(alkylamino)propionamides, and this compound may also trap another acrylamide molecule to produce a new adduct. Although 3-(alkylamino)propionamide was not stable and the reaction was reversible by heating, the activation energy required for the formation of 3-(alkylamino)propionamide was lower than the elimination reaction of the Michael adduct. Therefore, it was reported that acrylamide disappeared when it was stored in the presence of glycine at 60 °C for 14 days. However, when the samples were heated again at 180 °C for 20 min, a significant amount of acrylamide was detected.^87^ It could be possible that acrylamide could also be inhibited in the presence of amine sources in food products.

### Effect of Dough Conditions and Nixtamalization

The water activity, pH, and fermentation of dough can affect acrylamide formation in bakery products.^88^ For example, Jung et al.^88^ reported 82 and 73% reduction in the acrylamide content of fried and baked corn chips after 0.2% citric acid treatment, respectively. Nixtamalization of corn was reported to have a reducing effect on the formation of acrylamide in tortilla chips prepared with nixtamalized corn flour. For example, Ca(OH)_2_ at a concentration of 1.5 and 2.0 g/100 g reduced acrylamide by 52 and 36%, respectively, in tortilla chips compared to the chips prepared from the flour nixtamalized at a concentration of 1.0 g/100 g. In spite of the increase in the pH of the dough from 7.18 for 1.0 g Ca(OH)_2_/100 g to 8.50 and 8.71 for 1.5 g Ca(OH)_2_/100 and 2.0 g Ca(OH)_2_/100 g, respectively, a significant reduction could be achieved in the presence of calcium.^5^ Although the increase in pH toward alkaline conditions favors acrylamide formation,^89^ calcium from the nixtamalization process was able to limit the formation of acrylamide during heating. Further studies also confirmed the effect of nixtamalization of corn flour on acrylamide mitigation.^90^ The results of these studies indicated that an optimized nixtamalization process, which is conventionally applied, is an efficient way of reducing the acrylamide forming potential of corn flour. However, more studies should be conducted to evaluate the sensory changes when the amount and type of alkali agents are changed in this regard.

### Effect of Processing Conditions

In general, the baking and frying time and the temperature are considered to be the most critical processing factors affecting acrylamide formation in corn-based thermally processed foods.^36,88^ The type of frying oil can also be important in terms of the acrylamide reduction of corn-based products. Salazar et al.^91^ reported a 77% reduction in the acrylamide content of tortilla chips fried in piquin pepper oleoresin compared to tortilla chips fried in soybean oil. Optimization of different processes such as infrared heating, extrusion cooking, or microwave heating can also be helpful in the reduction of acrylamide in corn-based products. The acrylamide concentration in corn subjected to infrared heating at 140 °C for 100 s was reported to be 704 ng/g. It was approximately 4.5 times higher than in corn infrared heated at 110 °C for 50 s.^24^ The acrylamide levels of corn extrudates decreased by the increase in feed moisture regardless of the formulations. An 82% acrylamide reduction was achieved by increasing the feed moisture content from 22% to 24% with the combined effect of CO_2_ injection.^92^ Extrusion of corn snacks having 5% nanofiltered whey powder with a high food moisture (14%) was suggested to be better in terms of the acrylamide content and from a nutritional point of view.^93^ Moreover, the acrylamide contents were reported to be higher by 49.5–74.3% in corn products after vacuum microwave treatment for 10 min compared to infrared heating for 10 s.^94^ Thermal processing conditions also have an impact on the color of the products. In thermally processed foods, acrylamide formation takes place in parallel with browning, and therefore, measurement of color was used as an indication of acrylamide formation as well as the intensity of the thermal process in foods such as French fries, chips, and biscuits.^95−97^ All these studies identified the chromatic parameter a* as a useful predictor of acrylamide formation. In addition, Mesias et al.^97^ used the color parameter a* to discriminate French fries according to their acrylamide contents as “below” or “above” the benchmark level indicated as 500 μg/kg for fried potatoes by the EU regulation. A value of 0.855 for a* was found as the threshold value for acrylamide contents above the benchmark level. However, there is still no data about the correlation between acrylamide content and color in corn-based snacks. Such a correlation could be practically used for minimizing acrylamide exposure, both in household applications and by producers of corn-based products. Widely consumed thermally processed corn-based foods such as tortilla/corn chips, cornflakes, breakfast food, popcorn, different kinds of corn-based cookies/biscuits, snack foods, and bread are an important acrylamide source in the common diet of all age populations. However, as research has shown, higher intake values occurred among younger generations (toddlers, other children, adolescents, and young adults). With this in mind, the mitigation strategies should be applied in order to reduce the content of acrylamide in corn-based food products. Among the mitigation strategies, controlling asparagine by applying suitable crop regime management in the field would be one of the most effective methods because low asparagine corn could be used both in industry and in culinary applications. However, crop regime management is not very easy to apply as it requires season-based long-term tracking and measurements. Although using additives such as divalent cation salts or changing the conditions of dough could seem easier to apply, it may cause some undesirable textural or sensorial changes in the product as in the case of changing processing conditions such as thermal treatment temperature and time. Moreover, combined applications could require extra costs, time, and/or energy. For these reasons, it is necessary to choose the most realistic approaches for effective mitigation of acrylamide in corn-based products.

## Methodology

Search engines such as Google, ResearchGate, and especially KoBSON (scientific information service of the National Library of Serbia) were used to find literature sources and necessary information for this review paper. The analysis of literature sources was not timed, although special attention was paid to key information from the last 10 to 15 years. In the search for information, keywords such as corn production, corn-based food products, tortillas, popcorn, corn flakes, corn bread, corn nut, corn-based snacks, consumption of corn-based processed foods, free asparagine in corn grain, sugars in corn grain, the effect of crop management regimes on the chemical composition of corn grain, acrylamide in corn-based food products, acrylamide in cereal-based food products, human exposure to acrylamide from corn-based food products, recommended level of acrylamide, benchmark levels of acrylamide, acrylamide mitigation strategies, etc. were used.

## Abbreviations Used

Asn: asparagine
EC: European Commission
EFSA: European Food Safety Authority
Fru: fructose
FAO: Food and Agriculture Organization
Glc: glucose
IRdS: index of reducing disaccharides
IRmS: index of reducing monosaccharides
IRS: index of reducing sugars
LOD: limit of detection
LOQ: limit of quantitation
Mal: maltose
Suc: sucrose
TRS: total reducing sugars
TS: total sugars
